# Immunity in Protochordates: The Tunicate Perspective

**DOI:** 10.3389/fimmu.2017.00674

**Published:** 2017-06-09

**Authors:** Nicola Franchi, Loriano Ballarin

**Affiliations:** ^1^Department of Biology, University of Padova, Padova, Italy

**Keywords:** tunicates, immune responses, complement, lectins, inflammation, chemical defense

## Abstract

Tunicates are the closest relatives of vertebrates, and their peculiar phylogenetic position explains the increasing interest toward tunicate immunobiology. They are filter-feeding organisms, and this greatly influences their defense strategies. The majority of the studies on tunicate immunity were carried out in ascidians. The tunic acts as a first barrier against pathogens and parasites. In addition, the oral siphon and the pharynx represent two major, highly vascularized, immune organs, where circulating hemocytes can sense non-self material and trigger immune responses that, usually, lead to inflammation and phagocytosis. Inflammation involves the recruitment of circulating cytotoxic, phenoloxidase (PO)-containing cells in the infected area, where they degranulate as a consequence of non-self recognition and release cytokines, complement factors, and the enzyme PO. The latter, acting on polyphenol substrata, produces cytotoxic quinones, which polymerize to melanin, and reactive oxygen species, which induce oxidative stress. Both the alternative and the lectin pathways of complement activation converge to activate C3: C3a and C3b are involved in the recruitment of hemocytes and in the opsonization of foreign materials, respectively. The interaction of circulating professional phagocytes with potentially pathogenic foreign material can be direct or mediated by opsonins, either complement dependent or complement independent. Together with cytotoxic cells, phagocytes are active in the encapsulation of large materials. Cells involved in immune responses, collectively called immunocytes, represent a large fraction of hemocytes, and the presence of a cross talk between cytotoxic cells and phagocytes, mediated by secreted humoral factors, was reported. Lectins play a pivotal role as pattern-recognition receptors and opsonizing agents. In addition, variable region-containing chitin-binding proteins, identified in the solitary ascidian *Ciona intestinalis*, control the settlement and colonization of bacteria in the gut.

## Introduction

Tunicates or urochordates are marine, filter-feeding invertebrates, members of the phylum Chordata. They owe their name to the tunic that embeds the larval and adult body. Tunicates (*ca* 3,000 species) include Ascidiacea (benthic and sessile), Thaliacea (pelagic), and Larvacea or Appendicularia (pelagic).

Ascidians have a free-swimming, tadpole-like larva whereas adults have sac-like bodies with two siphons, allowing water flux, and a large branchial basket provided with a ventral endostyle secreting the mucous net required for filtration. They include Phlebobranchia, Aplousobranchia, and Stolidobranchia, previously grouped as Enterogona (Phlebobranchia and Aplousobranchia) and Pleurogona (Stolidobranchia).

**Figure 1:**
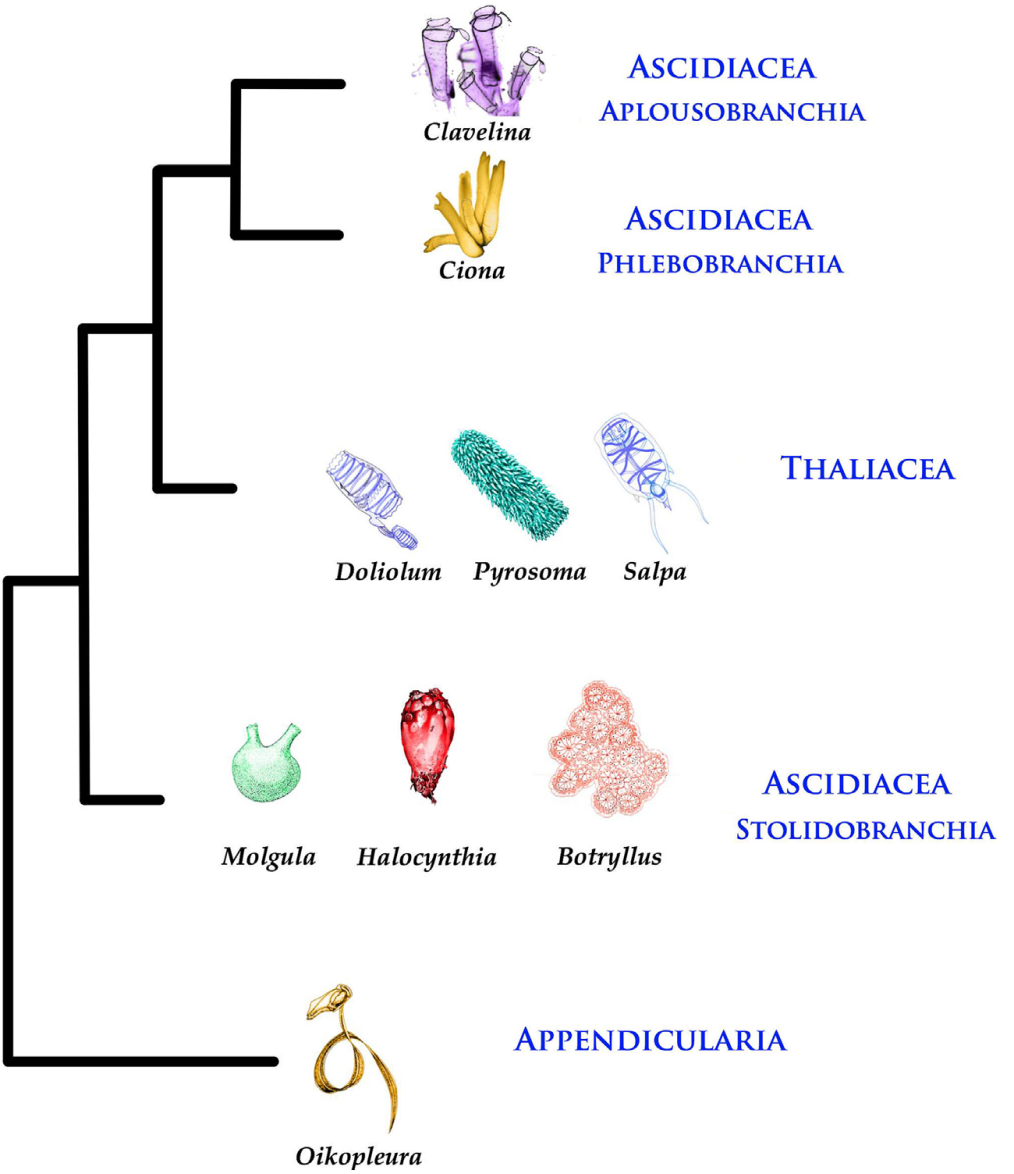
Phylogenetic tree of Tunicates [according to Ref. (1)].

Thaliaceans have barrel-like bodies; they include Pyrosomida (colonial), Doliolida (solitary/colonial), and Salpida (solitary/colonial). All Thaliaceans but Doliolida are devoid of larval stages. Larvaceans or appendicularians are similar to ascidian larvae, hence their name: they secrete a gelatinous house containing traps for food particles and use their tail to move water for filtration. Today, Larvaceans are considered a sister group of the remaining tunicates and Thaliaceans as a sister group of Enterogona (Figure 1).

Tunicates are the closest relatives to vertebrates (2), and this explains the increasing interest toward this group of animals. Like other invertebrates, tunicates rely only on innate immunity that lacks somatic recombination and long-term immune memory and has a limited array of effector responses.

Ascidians include about 2,300 species and are the most studied tunicates. Accordingly, the majority of the information on tunicate immune responses comes from studies on these organisms. In addition, ascidian innate immune genes did not undergo the expansions reported in other invertebrate deuterostomes, such as amphioxus and sea urchin (3, 4). This review, then, will focus mainly on the ascidian strategies of immune defense. Where available, information on immune responses of pelagic tunicates will be added.

## The Sites of Immune Responses

The marine habitat contains 10^5^–10^6^ microbes/ml in the water column and much more in the sediments (5); the amount of viruses is 10 times higher (6). Tunicates, therefore, require an efficient immune system in order to prevent the risk of infections and select appropriate mutualistic bacterial strains for gut colonization (see below). The sites where the ascidian immune system is alerted by the contact with non-self molecules include the tunic, the hemolymph, and the digestive tract.

### Tunic

The tunic represents the first outpost against pathogens and parasites and its damage, as in the soft tunic syndrome, can lead the organism to death (7). It is mainly of epidermal origin and resembles the vertebrate connective tissue in consisting of an amorphous matrix containing fibers and interspersed cells (8). The tunic can contain spicules, acting as physical defense against predators and varying in morphology, size, and mineral content (8). Molecules with antibacterial and anti-inflammatory activity are usually present in the matrix (9, 10). The tunic fibrous components include tunicin, a cellulose-like polysaccharide, collagen, and elastin (8, 11). Intermediate filaments (12) and mucopolysaccharides (9) contribute to the structural integrity of the tunic. The outermost compact layer, known as the cuticle, is continuous with the tunic matrix and frequently presents minute protrusions or spines (8, 13–15).

Tunic cells derive from both the epidermis and the hemocytes that can enter the tunic in response to infections (8, 16, 17). Hemocytes include spreading and round phagocytes, always present, cytotoxic granulocytes, widely found, and other cell types in some particular taxa, such as net cells and cells storing acid or pigments (8, 17–21), all contributing to protect the organism from predators, pathogens, or parasites. Phagocytes ingest foreign cells having entered the tunic (17, 22), and tunic phagocytes are the main effectors of allorecognition in the colonial species *Aplidium yamazii* (23). Granulocytes frequently contain and release antimicrobial peptides (24) and the enzyme phenoloxidase (PO) (25). Bladder cells store acid that, once released, decreases the pH of the tunic, disinfects the wounds, and exerts antifouling activity (17, 26, 27); net cells allow the shrinkage of the tunic in wound areas (17). PO-containing granulocytes can contribute to tunic formation or regeneration *via* degranulation and release of tunichromes, likely fragments of DOPA-containing proteins, that, once oxidized, cross-link tunicin fibers (28, 29). Tunic phagocytes and net cells are present also in Thaliaceans, although their role in defense has been poorly investigated. In pyrosomes, the density of tunic cells is comparable to that of ascidians (30), whereas doliolids and salps have a lower number of cells in their tunic (14, 17, 21). Larvaceans or appendicularians have no tunic, but tunicin is present in their house, secreted by specialized portions of the trunk epithelium (31).

### Hemolymph

**Figure 2:**
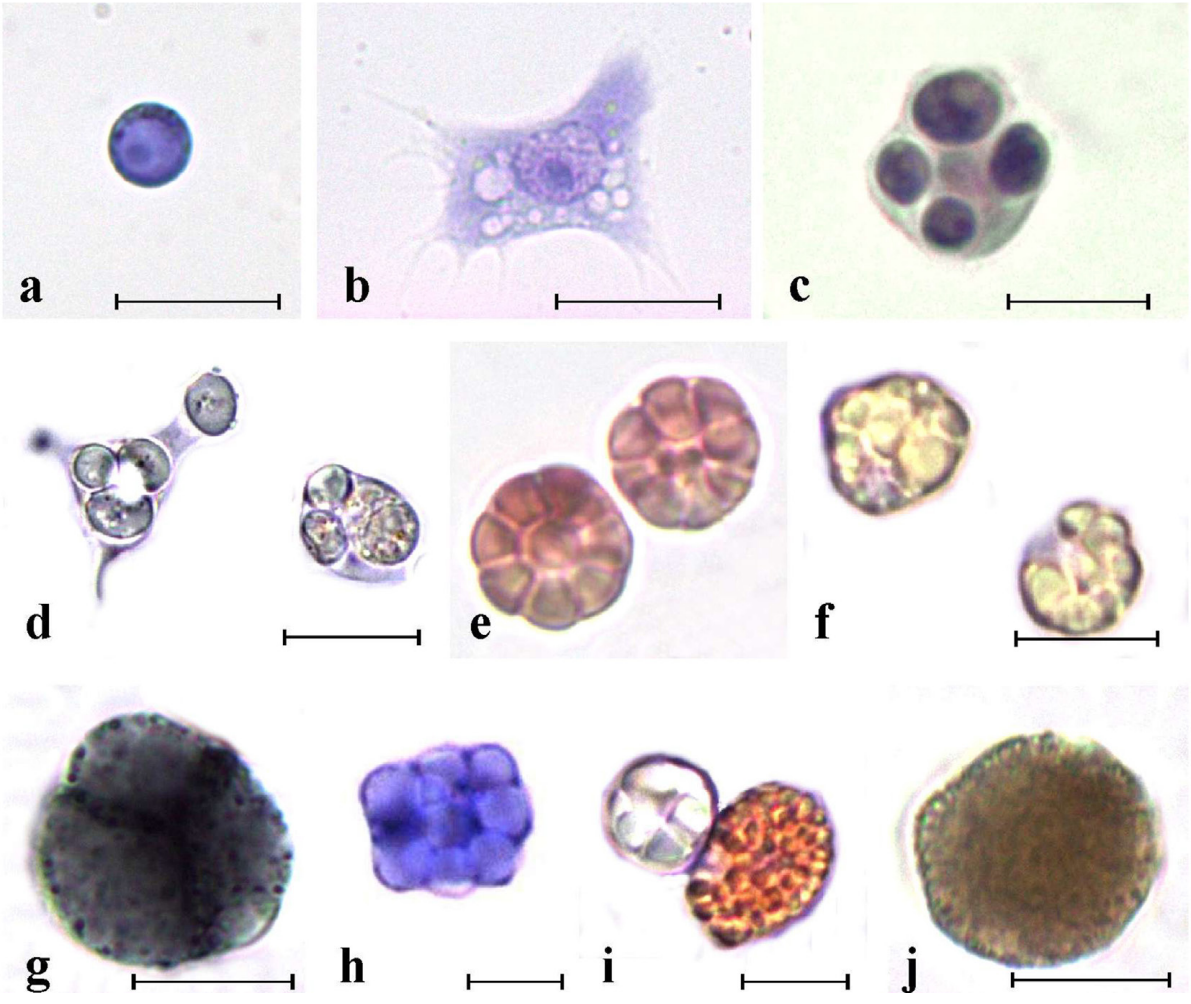
Main ascidian hemocytes. **(A)** Undifferentiated cells. **(B–F)** Immunocytes. **(B,C)** *Botryllus schlosseri* spreading and round phagocytes, respectively; **(D)** *Polyandrocarpa misakiensis* speading and round phagocyte with ingested yeast cells; **(E)** *B. schlosseri* morula cells (MCs); **(F)** *P. misakiensis* MCs; **(G–J)** storage cells. **(G)** *B. schlosseri* blue pigment cells; **(H)** *P. misakiensis* trophocyte; **(I)** *P. misakiensis* pigment cell and trophocyte; **(J)** *B. schlosseri* nephrocyte. **(A–C,E,H)** aldehyde-fixed cells stained with hematoxylin–eosin; **(D,F,G,I,J)** living cells. Scale bar: 10 µm.

Ascidians have an open circulatory system and a colorless hemolymph, isotonic with seawater. The beating of a tubular heart guarantees the circulation in blood sinuses and lacunae. It periodically reverses the direction of the peristaltic waves thus inverting the hemolymph flow (8, 13). Circulating hemocyte types differ in morphology and ultrastructure. Various authors proposed unifying classification schemes [Figure 2; Table 1; references therein (32)], but uncertainties and doubts persist on terminology, hemocyte relationships, and differentiation pathways.

**Table 1:** Ascidian main hemocyte and tunic cell categories.

	Cell types (synonyms)	Role	Reference
**Hemocytes**			
Undifferentiated cells	Hemoblasts (lymphocyte-like cells)	Considered hemocyte precursor cells	(8, 32)
Immunocytes	Phagocytes (hyaline amebocytes, macrophage-like cells, spreading and round phagocytes)	Phagocytosis; encapsulation; synthesis and release of lectins	(8, 13, 32, 33)
	Cytotoxic cells [phenoloxidase (PO)-containing cells, morula cells (MCs), granular amebocytes]	Cell-mediated cytotoxicity; synthesis and release of: cytokines, complement factors, antimicrobial peptides, and collagen	(8, 13, 32, 34, 35)
Storage cells	Pigments cells	Zooid pigmentation	(8, 13, 32)
	Trophocytes	Storage and transport of nutrients	(32)
	Nephrocytes	Storage of uric acid crystals	(8, 32)
	Vanadocytes	Storage of vanadium	(8, 32)
**Tunic cells**			
Immunocytes	Phagocytes	Ingestion of foreign material having entered the tunic; guard cells (external to the tunic and exposed to the environment in the siphonal areas) controlling the entrance to the pharynx or the atrium	(8, 17, 22, 23)
	Cytotoxic cells (PO-containing cells, MCs, granulocytes)	Cell-mediated cytotoxicity; synthesis and release of: cytokines, complement factors, antimicrobial peptides; crosslinking of tunicin fibers through the oxidation of tunichromes by PO	(8, 13, 24, 25, 28, 29)
Bladder cells		Acid storage	(17, 26, 27)
Net cells		Allow the shrinkage of the tunic in wound areas	(17)
Pigment cells		Tunic pigmentation	(17)

Ascidian hemocytes, involved in immune responses (immunocytes), represent a relevant fraction of circulating hemocytes (32), synthesize most of the pattern-recognition receptors (Table 2) and actively transcribe genes required for immune defense (60, 61): they include phagocytes and cytotoxic cells. Phagocytes are wandering, spreading cells that actively move toward foreign cells or particles and ingest them. Upon the ingestion of foreign material, phagocytes withdraw their projections and assume a round morphology. Spreading phagocytes can reach 20 µm in length and have a well-defined actin cytoskeleton, with abundance of stress fibers (25). They contain fine cytoplasmic granules, unresolvable under the light microscope, showing positivity for lysosomal enzyme activities (32). Round phagocytes are large cells (15–20 µm in diameter) with one or more phagosomes containing the ingested material as well as hydrolytic enzymes, lipids, and lipofuscins (32). In the colonial ascidian, *Botryllus schlosseri*, the presence of a static and a mobile population of phagocytes was described: the former adhere to the basal lamina of the peribranchial epithelium and form the ventral islands, on both sides of the endostylar sinus (62).

**Table 2:** Ascidian main pattern-recognition receptors.

Name	Location	Role	Reference
**Lectins**			
Galectins	Granulocytes, phenoloxidase (PO)-containing hemocytes	Mediators of inflammation	(36)
Rhamnose-binding lectins	Phagocytes	Phagocyte activation, opsonins, hemocyte recruitment, PO-containing cell degranulation	(37)
Sialic acid receptor (unknown)	Phagocyte surface	Recognition of sialic acid (do not eat me signal) on the surface of healthy cells	(38)
CD91	Surface of PO-containing cells	Indirect activation of phagocytes	(39)
**VCBPs**			
VCBP-A, -B, -C	Hemocytes, epithelial cells of stomach and intestine	Opsonins, control of the gut microbiota	(40, 41)
**Complement factors**			
Mannose-binding lectins	PO-containing hemocytes	Activation of the lectin pathway of complement activation	(42–49)
Ficolins	PO-containing hemocytes	Activation of the lectin pathway of complement activation	(49–52)
C1q	Presumably PO-containing hemocytes	Complement activation by binding to pentraxins	(53–55)
Integrins	Presumably phagocytes	Complement receptor(s)	(32, 46, 56, 57)
Toll-like receptors	Phagocyte surface and endosomes	Recognition of non-self	(48, 58)
CD36	Phagocyte surface	Recognition of oxidized lipids on apoptotic cells	(59)
Phosphatidylserine receptor (unknown)	Presumably phagocyte surface	Recognition of apoptotic cells	(38, 59)

Cytotoxic cells are granular cells, 10–15 µm in diameter; their cytoplasm is filled with large granules containing the inactive form of PO (34). They frequently constitute the most abundant circulating hemocyte type (32). In most of the studied species, cytotoxic cells assume a typical berry-like morphology after aldehyde fixation and are called morula cells (MCs).

As regards pelagic tunicates, Cima et al. (21) reported the characterization of circulating hemocytes of *Thalia democratica* oozooids: they include phagocytes that contain hydrolytic enzymes in their cytoplasm and can migrate into the tunic. Larvaceans have no hemocytes (21).

Hemocytes containing histamine and heparin inside their granules were observed in both ascidians and Thaliaceans: the molecules can either stabilize the granular content or, when released, modulate the inflammatory reaction by inducing tunic vessel-contraction and inhibition of phagocytosis (21, 63).

### Digestive System

The oral and the atrial (cloacal) siphons are preferential ways of entrance of microorganisms. Here, a population of phagocytes is exposed to seawater, adhering to the internal tunic. Such sentinel or guard cells can recognize and ingest foreign particles or cells, thus preventing their entrance in the pharynx or in the atrium (64); they were found also in Thaliaceans (21).

In the solitary ascidian *Ciona intestinalis*, both the endostyle and the gastric epithelium constitutively transcribe genes involved in the inflammatory response triggered by the injection of LPS in the body wall (11, 65, 66), suggesting the importance of the alimentary tract in the recognition and the clearance of non-self material. This assumption is corroborated by the reported transcription of genes for Toll-like receptors (TLRs), mannose-binding lectins (MBLs), and MBL-associated serine proteases (MASPs) in both the stomach and the intestine, in addition to hemocytes, in accordance with the important immunosurveillance role of the alimentary tract (48, 58). In addition, variable region-containing chitin-binding proteins (VCBPs), secreted in the gut lumen and recognizing the surface of Gram (+) and Gram (−) bacteria (see below), probably exert a pivotal function in the maintenance of a stable commensal gut microbial flora. This is consistent with the hypothesis of a role of the immune system in both protecting host tissues from pathogenic attack and supporting the growth of the mutualistic microbiota (67). In *B. schlosseri*, gut epithelial cells are involved in the clearance of neighboring apoptotic cells during the generation change (68).

## Humoral Defensive Repertoire

### Phenoloxidase

The presence of PO activity in ascidian hemolymph has been widely reported in both solitary and colonial species [references therein (34)]. PO is assumed located as inactive proenzyme (probably, proPO), inside the granules of PO-containing hemocytes and activated by serine proteases once released outside the cells (35, 69). PO-containing hemocytes of *C. intestinalis* store also serine proteases that, once released, are activated by LPS and laminarin shortly before the activation of PO (70, 71). A soluble serine protease is present also in *B. schlosseri* hemolymph (72). This support the idea of an activation of PO mediated by serine proteases, analogous to what is reported in arthropods (73).

Phenoloxidase is involved in cytotoxic responses of ascidians. In colonial botryllid ascidians, the enzyme contributes to the formation of the necrotic spots along the border of contacting, genetically incompatible colonies (34). According to the analysis of nucleotide and predicted amino-acid sequences, ascidian PO shows high similarity with arthropod hemocyanins (74, 75).

Phenoloxidase substrates are likely represented by tunichromes, or other phenol-containing peptides, contained inside the hemocyte (mainly MC) granules (29, 35, 76–79). The enzyme produce quinones, that polymerize to melanin, and reactive oxygen species (ROS), that induce oxidative stress and related toxicity in neighboring cells (35).

### Lectins

Ascidian immunocytes can synthesize and release humoral lectins with various molecular features and carbohydrate specificities (36, 80–84). Some of them have a clear role in the recognition of foreign molecules or in the modulation of immune responses (36, 65, 85–88). In most cases, they enhance the phagocytosis of microorganisms acting as opsonins (86, 87, 89, 90). Lectins can also trigger the respiratory burst and act as molecules able to influence the behavior of other immunocytes, as in the case of the *Botryllus* rhamnose-binding lectin (BsRBL) (37), or to activate the complement system (46).

A subset of *B. schlosseri* blood cells, probably phagocytes, express an ortholog of the vertebrate CD94 receptor on NK cells, a type II transmembrane protein with a C-type lectin domain (91). A second ortholog in *C. intestinalis* (CiCD94-1) contain a C-type lectin domain without carbohydrate-binding capability: it probably recognizes peptides instead of carbohydrates and is expressed in the same cell type engaged in the production of PO, also recognized by the anti-CiCD94-1 antibody. The fraction of cells positive to the CiCD94-1-1 antisense riboprobe increases after LPS exposure. The anti-CiCD91-1 antibody inhibits phagocytosis, suggesting that the interaction of CiCD94-1 with its ligand(s) can indirectly stimulate phagocytes (39, 92), probably through the release of cytokines (see below).

### Immunoglobulin (Ig) Domain-Containing Proteins

Despite the lack of orthologs of genes for major histocompatibility complex proteins, T-cell receptors, and Igs, transcripts for putative molecules with Ig domains were identified in tunicates (93, 94). Three novel genes for VCBPs, containing two N-terminal, variable-type Ig domains, were described in *C. intestinalis*. VCBP-A, -B, and -C are synthesized by epithelial zymogenic cells of the stomach and the intestine, as well as by a fraction of circulating hemocytes (40, 95). VCBPs can bind Gram (+) and Gram (−) bacteria with the variable-type Ig domains and significantly increase microbe phagocytosis by hemocytes, acting as opsonins (40), whereas the chitin-binding domain interacts with the chitin-rich mucus along the intestinal wall, thus influencing the settlement of bacterial communities and the colonization of the intestinal lumen by the microbiota. Indeed, VCBP-C can enhance the *in vitro* production of biofilms by bacteria previously identified in the gut of *Ciona* (41).

### Complement System

**Figure 3:**
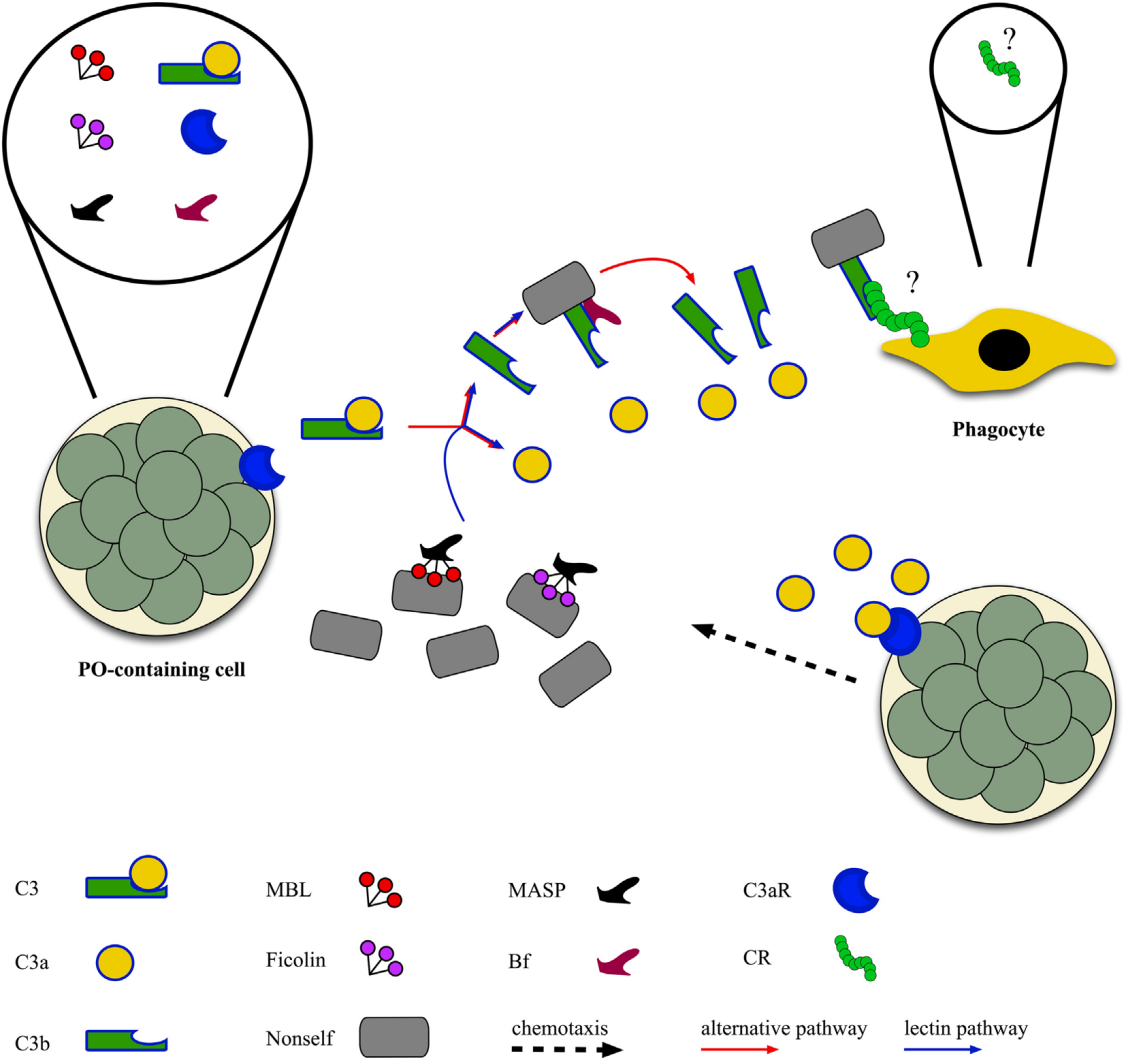
Ascidian complement-activation pathways: complement components of both the alternative and the lectin pathway [C3, Bf, mannose-binding lectins (MBLs), ficolins, MBL-associated serine proteases (MASPs)] are released by morula cells that also express the receptor for C3a (see text), whereas the receptor(s) for C3b [complement receptor (CR)] are probably located on the surface of phagocytes as the activation of C3 increases the phagocytic activity.

Both the alternative and lectin complement-activation pathways are present in ascidians [Figure 3; (46, 96)]. Genes for C3 were identified in all the ascidian species investigated so far (97–100). They are active in the adult (98), and their transcription rate increases after LPS injection in the tunic; similar behavior is reported for the C3-a fragment deriving from the cleavage of C3 in the presence of non-self (101). C3-a can recruit hemocytes to the inflammation site (102) *via* its binding to a G protein-coupled receptor, constitutively expressed in PO-containing hemocytes (103). C3b, the main C3 fragment, can adhere to the microbial surfaces and exert an opsonic role enhancing the recognition and ingestion of bacteria by phagocytes (89, 97, 98, 100, 104).

The transcription of C3 genes occurs in hemocytes, mainly PO-containing hemocytes (97, 100, 101). In *Styela plicata*, hemocytes secrete a protein recognized by anti-C3 antibodies, the concentration of which increases in the culture supernatant after the exposure to non-self molecules (105). In *Pyura stolonifera*, the incubation of hemolymph with LPS induces the release of a chemotactic protein recognized by anti-human C3 antibody (99). In *Halocynthia roretzi*, also cells of the stomach wall transcribe C3 (97), whereas, in *Ciona*, even ciliated cells bordering the branchial stigmata contain C3 mRNA (101).

Transcripts for Bf, a component of the alternative activation pathway, were identified in various ascidian species (100, 106, 107). Genes for MBLs, C-type lectins members of the collectin family and involved in the lectin pathway of complement activation, are present in the *C. intestinalis* genome (42, 44, 46–48) and over-transcribed during inflammatory reactions (42). Transcripts for MBLs were identified also in other ascidian species (43, 45, 49). In *S. plicata* (108, 109), an increase in the secretion of collectins and in the fraction of hemocytes immunopositive to anti-collectin antibody is observable during inflammatory responses (110). Transcripts for ficolins, also components of the lectin pathway, are present in *H. roretzi* (50), *Botrylloides leachii* (51), and *B. schlosseri* (49, 52). The transcription of *H. roretzi* ficolin 3 gene is significantly impaired in organisms with the soft tunic disease (7). A C-type lectin, interacting with MASP, is involved in the recognition of microbial surfaces and the activation of C3 in *H. roretzi* (111). Transcripts for MASPs were widely described in ascidians (43–46, 48, 49, 55, 96, 104, 112, 113).

C1q-like transcripts were found in *C. intestinalis* (53, 54) and *B. schlosseri* (55). In vertebrates, C1q, a component of the classical activation pathway, can bind pentraxins (mainly C-reactive protein). These molecules were identified in *Ciona* (53) and *Didemnum candidum* (83), suggesting the interaction with pentraxins as the original role of C1q in invertebrate chordates (53). In *B. schlosseri*, the transcription of genes for C1q, MASPs, Bf, and ficolins is upregulated during the allorejection reaction (55); in addition, genes for C3, Bf, ficolin, MASPs, and a putative CR1 are over-transcribed during the recurrent generation changes (113).

As regards complement regulators, in *B. schlosseri*, cDNAs for a putative complement-control protein (CCP), featuring CCP domains, were isolated (114). Genes for α2-macroglobulin, able to inhibit MASPs, and for various putative molecules with the CCP domain(s), were reported in *C. intestinalis* (44).

C6/C9-like transcripts for proteins containing the membrane-attack complex/perforin domain were described in *C. intestinalis* (44, 46, 47); whether or not a cytolytic pathway is present ascidians, is still a matter of debate.

In *Ciona*, integrin α and β subunits, part of a complement receptor (CR) and showing homology with mammalian CR3 or CR4, are expressed on the surface of hemocytes (46, 56, 57).

### Chemical Defense

Ascidians are the source of a great variety of bioactive molecules of potential interest in the sanitary field; some of them have also entered human clinical trials (115). Many compounds act as antiviral or repellents against foulants, predators, and competitors (116–120). Acid substances and metals stored in vacuoles within tunic cells can contribute to additional protection (26, 121, 122). The tunic may host prokaryotes that produce many of the above-reported products (115, 121).

Ascidians produce also molecules with antimicrobial activity (123–126). Most of them are peptides; in many cases, they are synthesized by hemocytes, mostly PO-containing cells. In *H. roretzi*, halocyamines A and B are synthesized by MCs (127), and their cytotoxic activity is likely related to the presence of diphenol rings that render them substrates for PO. *S. clava* MCs produce clavanins A–D, histidine-rich, α-helix peptides, and clavaspirin (128, 129). In the same species, five styelins, cationic antimicrobial peptides, were identified and isolated from hemocyte lysates (130, 131). In *C. intestinalis*, PO-containing hemocytes synthesize two families of α-helix antimicrobial peptides and the injection of non-self material in the body wall enhances the transcription of the corresponding genes (24, 132–134). Anticancer derivatives were also described (135, 136), and ascidian tunichromes can exert a cytotoxic activity (28). A gene homologous to mammalian EB1, a protein with tumor suppressing effect, was described in *B. schlosseri* (137).

### Cytokines and Cross Talk between Immunocytes

Despite the common opinion that invertebrate cytokines share no homologies with their vertebrate counterparts (138, 139), putative genes for IL1 and TNF receptors were identified in the *Ciona* genome (44, 61). A gene for a TNFα homolog, the transcription of which increases in *Ciona* hemocytes after LPS injection in the body wall, was also cloned (11, 140): it probably exerts a role in recruiting hemocytes to the inflamed area (141). Genes for a putative IL17 receptor and three IL17 homologs were identified in *Ciona* (3, 60, 61): their expression (in hemocytes) is also upregulated after LPS injection in the tunic (142).

**Figure 4:**
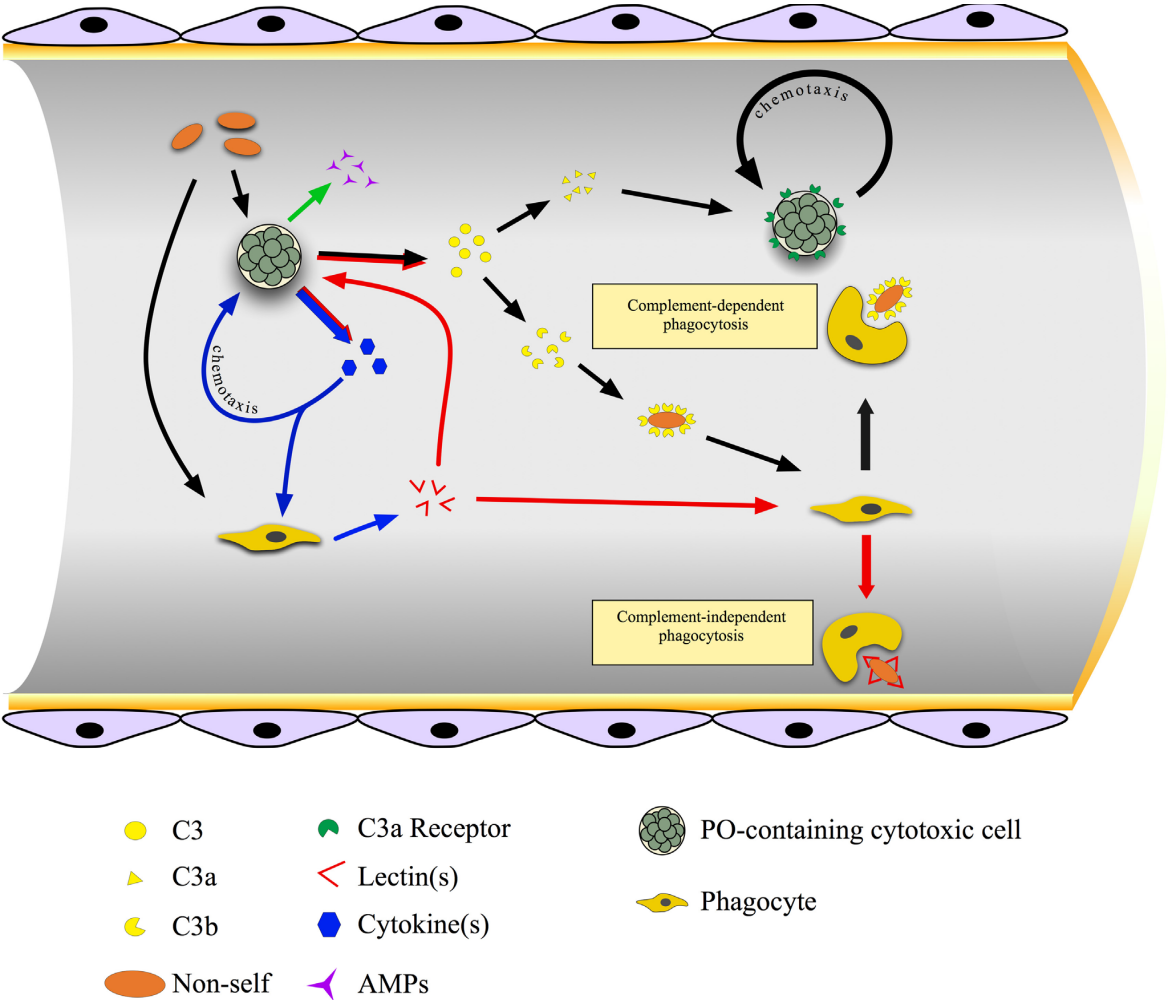
Cross talk between immunocytes in the colonial ascidian *Botryllus schlosseri*. Cytotoxic, phenoloxidase (PO)-containing cells are the first cells to sense non-self material and, as a consequence, they synthesize and release cytokines, antimicrobial peptides, and complement C3. Cytokines act on both morula cells (MCs) themselves, inducing their chemotaxis, and on phagocytes triggering the synthesis and the release of lectins, mainly rhamnose-binding lectin (RBL), that bind carbohydrates on the microbial surfaces and exert a complement-independent opsonic role. C3 is cleaved to C3a, which cooperates in recruiting MCs, and C3b, which interacts with the microbial surface and acts as opsonin.

In *B. schlosseri*, MCs are the main source of molecules recognized by antibodies raised against mammalian pro-inflammatory cytokines, secreted upon the recognition of foreign molecules (143). They induce phagocytes to synthesize and release BsRBL, with opsonic activity [Figure 4; (144)]. Anti-cytokine antibodies prevent the increase in phagocytosis observed when hemocytes are incubated in the supernatants of hemocytes cultures previously challenged with yeast (*Saccharomyces cerevisiae*) cells (145). In botryllid ascidians, during the allorejection reaction, MCs produce and release molecules immunopositive to anti-IL1α and anti-TNFα antibodies (25, 100, 146). They are involved in the recruitment of these cells to the ampullae of the contact region (see below), as demonstrated by the inhibition of the MC chemotaxis, induced by cell-free hemolymph from incompatible colonies, in the presence of the above-reported antibodies (146, 147). In *B. schlosseri*, the gene for an IL-17 ortholog is over-transcribed during the generation change: it probably modulates the cellular events occurring during this phase of the colonial life cycle and mediates the cross talk between MCs and phagocytes (113). A cooperation between MCs and phagocytes was reported also in *C. intestinalis* (70).

## Variety of Cell-Mediated Immune Responses in Ascidians

### Hemocyte Aggregation

Tunicate lack a coagulation system and hemocytes migrate and aggregate to plug the injured sites and prevent hemolymph leakage. Hemocyte aggregation was particularly studied in the solitary ascidian *H. roretzi* (148) where a membrane glycoprotein, active in both phagocytosis and hemocyte aggregation was identified (149). It contains two immunoreceptor tyrosine-based activation motifs (ITAMs) and associates with phosphorylated and unphosphorylated proteins, strongly suggesting its involvement in triggering signal transduction pathways (150). Further analyses demonstrated that, during hemocyte aggregation, it induces gene transcription through the activation of phosphatidylinositol-3 kinase (PI3K) and cytosolic calcium rise (151).

### Endocytosis

In ascidians, the ingestion of foreign materials occurs through either macropinocytosis or phagocytosis. In both cases, integrins and molecules containing the Arg–Gly–Asp (RGD) motif (e.g., fibronectin or fibrinogen) are involved (25). Pattern-recognition receptors allow the direct interaction of circulating professional phagocytes with potentially pathogenic foreign material. As an alternative, they recognize opsonins covering the microbial surfaces and enhancing phagocytosis. Opsonin-mediated phagocytosis can be either complement-dependent or complement-independent (Figure 4). A transient rise in cytosolic Ca^2+^ concentration is required for the ingestion, whereas a sustained increase lowers the extent of phagocytosis (25). The interaction of phagocytes with non-self particles triggers a respiratory burst, with the activation of both a membrane oxidase and an inducible nitric oxide (NO) synthase that leads to the production of ROS and reactive nitrogen species with microbicidal activity (152).

As for receptors involved in endocytosis, in *C. intestinalis*, two TLR genes were identified (60) and fully characterized: the corresponding proteins have cytoplasmic TIR, transmembrane, and extracellular LRR domains and are located in both the plasma membrane and the endosome membrane of phagocytes (58). In addition, *Ciona* also possesses a rich repertoire of transcripts of genes involved in signal transduction, including those for proteins with immunoreceptor tyrosine-based inhibition motifs and ITAMs, MyD88, IL1 receptor-associated kinase, TNF receptor-associated factor, nuclear factor κB (NF-κB), and inhibitor of κB (44, 53, 60). In the colonial *B. schlosseri*, TLRs are present on the surface and the interior of phagocytes (25). Here, the signal transduction pathways triggered by non-self recognition, include the activation of trimeric G-proteins, protein kinase A, protein kinase C, PI3K, mitogen-activated protein kinases (MAPKs), and NF-κB (25, 153, 154).

Phagocytosis of apoptotic cells is a common event in botryllid ascidians, where cyclical generation of new zooids by budding occurs, and old zooids are periodically resorbed (155). Generation change or take-over implies massive apoptosis in the tissues of old zooids and the clearance of dying cells by professional and occasional phagocytes (68, 156–158). Phagocytes recognize phosphatidylserine and the lack of sialic acid on the surface of effete cells and corpses (38, 59) and avidly ingest them: because of the sudden increase of oxygen consumption and the related oxidative stress, they undergo phagocytosis-induced apoptosis and are, in turn, ingested by other phagocytes (159). Clearance of dying cells requires also the presence of CD36, a scavenger receptor able to recognize oxidized lipids, on the phagocyte surface (59); a putative CD36 ortholog was identified in the *Ciona* genome (44). In *B. schlosseri*, the clearance of apoptotic cells by phagocytes is necessary for the completion of the take-over and the progression of bud development (160). The opposite is also true: buds are required for the clearance of cell corpses as they recycle the nutrients deriving from their digestion by phagocytes (161, 162).

### Encapsulation

Foreign material too large to be ingested by phagocytosis is usually encapsulated by circulating hemocytes. The formation of multi-layered capsules was observed around parasitic crustaceans, and both phagocytes and cytotoxic MCs can be involved in capsule formation (33). In *C. intestinalis*, intratunical injection of mammalian erythrocytes or non-self molecules results in massive recruitment of hemocytes to the inoculum site and capsule formation (11).

In *B. scalaris*, unlike other botryllid ascidians (see below), encapsulation plays a pivotal role in allorecognition. Here, the circulatory systems fuse during allorejection and blood exchange begins. Phagocytes crowd inside the fused vessels and stimulate the aggregation of hemocytes into large clusters, finally encapsulated by other phagocytes, so to plug the lumen of the vessels and interrupt the hemolymph flow in a few minutes (163).

### Cytotoxicity

A Ca^2+^-dependent cytotoxic activity against mammalian erythrocytes or tumor cells, inhibited by sphingomyelin, was described in *C. intestinalis* and *S. plicata* (164–166). In *C. intestinalis*, cytotoxicity against mammalian cells requires the activity of the enzyme phospholipase A2, modulated by lectins with specificity for galactosides (167). A cytotoxic reaction, called *contact reaction*, occurs in allogeneic or xenogeneic combinations of hemocytes from various solitary ascidians (168). In *B. schlosseri*, cytotoxicity can be observed *in vitro* by exposing hemocytes to non-self molecules or cell-free hemolymph of incompatible colonies (79). In all the above cases, cytotoxicity is consequent to the release of active PO in the medium upon degranulation of PO-containing hemocytes and the oxidation of polyphenol substrata, leading to the production of toxic quinones and ROS (34). In *B. schlosseri*, NO is also involved in the induction of cell death (146). The production of NO by hemocytes, after their exposure to either LPS or zymosan, was reported also in *S. plicata* and *Phallusia nigra* (169, 170).

### Inflammation

Inflammation is characterized by the recruitment of circulating hemocytes, extravasation, cell degranulation, induction of cytotoxicity, and phagocytosis (or encapsulation) of the foreign material. Inflammation-related cytotoxicity requires the recruitment of PO-containing hemocytes and the release of active PO in the infected area (142, 171, 172). It was particularly studied in *C. intestinalis*, after the injection of foreign material in the tunic (11). Circulating hemocytes of treated animals increase the transcription of genes involved in the recognition of non-self and tissue repair (11, 173–175).

#### Inflammation in Tissue Transplantation

Tissue transplantation represents a cause of inflammation. In solitary species, higher recruitments of hemocytes occur in the case of allografts with respect to autografts, leading to allograft rejection. The latter is more rapid in primed animals, having previously received (and rejected) a similar graft (176–178). Graft rejection relies on PO-containing hemocytes reaching the inflamed area and the induction of cytotoxicity (179). In *C*. *intestinalis*, the products of a polymorphic gene, structurally similar to a vertebrate CR and containing CCP domains, were proposed as individuality markers. They are synthesized by hemocytes, with various splice variants and high interindividual variability (180, 181).

#### Inflammation in Allorecognition

**Figure 5:**
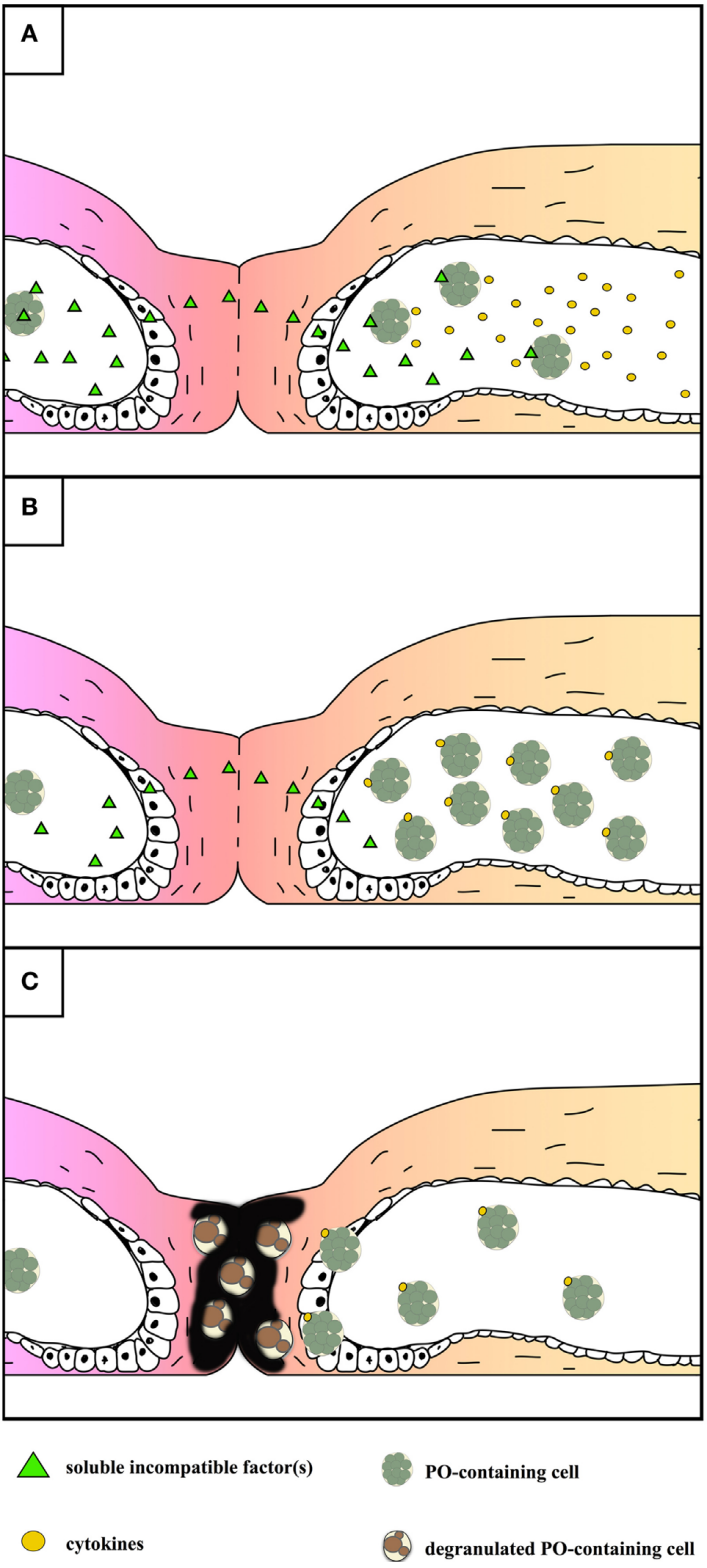
Schematic representation of the events occurring during the allorejection reaction of *Botryllus schlosseri*. For sake of simplicity, the main steps are reported on the right colony only. **(A)** local fusion of the contacting tunics and diffusion of soluble, incompatible factor(s) recognized by morula cells (MCs) inside the facing ampullae of the alien colony that, as a consequence, release cytokines. **(B)** Recruitment of MCs inside the tips of the ampullae facing the alien colony. **(C)** Extravasation of MCs and their degranulation in the tunic: melanin is formed as a consequence of the release of polyphenols and active phenoloxidase (PO); both melanin and reactive oxygen species contribute to the cytotoxicity observed in the contacting region.

In botryllid ascidians, inflammatory events are the consequence of allorecognition between incompatible colonies, probably to prevent the risk of somatic/germ cell parasitism in genetically unrelated colonies (182, 183). In *Botryllus primigenus* and *B. schlosseri*, a highly polymorphic fusibility/histocompatibility (Fu/HC) gene with codominant alleles controls the outcome of the colony contact (184, 185). When colonies share no alleles at the Fu/HC locus, partial fusion of the facing tunics occurs as well as the leakage of soluble histocompatibility factors, recognized by MCs (186). Activated MCs release chemotactic cytokines able to recruit other MCs in the peripheral blind endings of the tunic vasculature (ampullae) of the contact region (147), from which they enter the tunic and degranulate, thus releasing the enzyme PO and its polyphenol substrata. A series of melanic cytotoxic foci, called points of rejection, appear along the contact border as a result of cytotoxicity [Figure 5; (34, 79, 146, 187)]. Rejecting colonies of *B. schlosseri* increase the transcription rate of various immune-relevant genes (52, 55, 188). A change in the growth direction of contacting colonies occurs after the allorejection reaction (189). MCs are involved in the allorejection reaction also in *Botrylloides simodensis, Botrylloides fuscus, Botrylloides violaceus, B. leachii* (35), and *Didemnum perlucidum* (190). The unusual growth of facing ampullae during allorecognition was reported in *B. leachi* (35, 191).

An intense inflammatory reaction is observed when incompatible colonies of ovoviviparous botryllid ascidians are brought into contact at their cut surfaces (192, 193), whereas fusion of tunics and hemolymph vessels always occurs in the case of viviparous species. This suggests that, in the latter case, hemocytes have lost their ability of allorecognition, probably to avoid immune attacks toward the brooded embryos that share only one Fu/HC allele with the mother colony (194). In support of the above hypothesis, the PO activity of the hemolysate of viviparous species is lower than that of ovoviviparous ones (195, 196).

When *Botryllus* colonies share at least one allele at the Fu/HC locus, contacting colonies can fuse and form a single chimeric colony (197). However, in the case of a single shared allele, the resorption of one of the chimeric partner occurs within 30 days from the temporary fusion (198). Even in this case, MCs are directly involved as they infiltrate the tissues of the loser colony, together with phagocytes. The resorption phenomenon can be induced by the injection of enriched populations of MCs in the vasculature of recipient colonies and shares many similarities with the take-over, including apoptosis in zooid tissues, clearance of dying cells by phagocytes, and modulation by IL17 (113).

In *B. schlosseri*, the ampullar epithelium and hemocytes express genes for proteins involved in allorecognition, although uncertainties on the identity of the allorecognition gene still persist (199–204).

## Role in Development?

Many invertebrate molecules have a role in both development and immunity. The best example is the *Drosophila* Toll receptor, required for the establishment of dorsal–ventral polarity early in development and switching to an immune role in adult flies (205). In Tunicates, various genes, involved in adult immune responses, are transcribed also during embryonic, larval, and asexual development, and this opens interesting perspectives on their role in development.

In *B. villosa*, the analysis of the transcriptome revealed the expression of immune-related genes in both the larval and juvenile development (43). In addition, MASPs are probably involved in the activation of metamorphosis (206).

In the larva of the ascidian *Ascidia callosa*, tunichrome, the putative substrate of PO, is required for tunic morphogenesis (207).

In *C. intestinalis*, a C3-like gene is transcribed during early development: it codifies a protein that, probably, does not exert a typical C3 role (208). Orthologous genes of C6 and C1 are also active during the embryonic stage (54). In addition, the gene for CiCD*94-1* is transcribed in larval papillae, in cells of the larval nervous system, and in the coronet cells, the probable precursors of neural crest cells, with a role in modeling the nervous system during development (39). Furthermore, swimming larvae transcribe a gene for a CiTNFα-like protein (141), and PO gene expression is modulated in early and larval development (209). In the same organism, very low transcription levels of VCBP genes can be detected before the tailbud stage. From the larval stage onward, their mRNAs are located in gut primordia, with different distributions in defined territories, suggesting a role of VCBPs in the functional compartmentalization of the developing intestine (95, 210). VCBP mRNAs are translated after metamorphosis, with different timing of appearance and distribution (41). The transcription of VCBP genes in juveniles is differentially modulated by Gram (+) and Gram (−) bacteria, fitting the idea of their role in mediating the onset of the microbial gut colonization (95, 210).

An increase in the transcription of several immune-related genes occurs also during the whole body regeneration of *B. leachii* (51). In addition, signaling pathways, such as those involving MAPK and the NF-κB/Rel family members, are required in the formation of the larval notochord (211) and in the budding process of botryllid ascidians (212).

## Future Perspectives

Tunicates, and ascidians in particular, are simple chordates that represent valuable models for the study of the innate immune responses and the evolutionary events that occurred in the course of invertebrate–vertebrate transition, leading to the appearance of lymphocytes and receptor diversification through somatic recombination. The progressive availability of new sequenced transcriptomes and genomes from tunicates will enable researchers to dissect the genetic and molecular processes associated with immune responses, clarify the regulatory pathways and the diversity of pattern-recognition receptors involved in immune responses, and compare them with what known in vertebrates. Ascidians offer also the possibility to study some particular aspects of the immune responses, such as the evolutionary importance of the polymorphism found in Fu/HC and other immune genes and its relationships with pathogen threats, the molecular basis of the priming phenomenon, the evolution of the complement system, and the role of lectins as immunomodulatory molecules. In addition, the possibility of synthesizing the gene products once the gene sequences are known, can render available a quantity of bioactive molecules, involved in chemical defense, testable as antimicrobial, antiviral, or anticancer compounds. Last, but not least, research on hemocytes will contribute to disentangle the unresolved aspects of hemocyte ontogeny and differentiation pathways and better elucidate their role in tunicate biology.

## Author Contributions

LB set up the work plan. LB and NF equally contributed to the text of the review.

## Conflict of Interest Statement

The authors declare that the research was conducted in the absence of any commercial or financial relationships that could be construed as a potential conflict of interest.
